# Imperfect Predictors for Lung Cancer Immunotherapy—A Field for Further Research

**DOI:** 10.3389/fonc.2020.568174

**Published:** 2020-11-30

**Authors:** Kamila Wojas-Krawczyk, Tomasz Kubiatowski

**Affiliations:** ^1^ Department of Pneumonology, Oncology and Allergology, Medical University of Lublin, Lublin, Poland; ^2^ Department of Clinical Oncology, Saint John of Dukla Oncology Centre of the Lublin Region, Lublin, Poland

**Keywords:** predictive factors, PD-L1, PD-1, immunotherapy, non-small cell lung cancer

## Abstract

The armamentarium for lung cancer immunotherapy has been strengthened using two groups of monoclonal antibodies: 1) anti-PD-1 antibodies, including pembrolizumab and nivolumab, which block the programmed death 1 receptor on the lymphocyte surface, resulting in increasing activity of these cells, and 2) anti-PD-L1 antibodies, including atezolizumab, durvalumab, and avelumab, which block the ligand for the PD-1 molecule on tumor cells and on tumor-infiltrating immune cells. The effectiveness of both groups of antibodies has been proven in many clinical trials, which translates into positive immunotherapeutic registrations for cancer patients. Regarding the predictive factor, PD-L1 expression on cancer cells is the only biomarker validated in prospective clinical trials used for qualification to immunotherapy in advanced non-small cell lung cancer (NSCLC) patients. However, it is not an ideal one. Unfortunately, no clinical benefits could be noted in patients with high PD-L1 expression on tumor cells against the effectiveness of immunotherapy that may be observed in patients without PD-L1 expression. Furthermore, the mechanism of antitumor immune response is extremely complex, multistage, and depends on many factors. Cancer cells could be recognized by the immune system, provided tumor-specific antigen presentation, and these arise as a result of somatic mutations in tumor cells. Based on novel immunotherapy registration, high tumor mutation burden (TMB) has become an important predictive factor. The intensity of lymphocyte infiltration in tumor tissue may be another predictive factor. The effectiveness of anti-PD-L1 immunotherapy is observed in patients with high expression of genes associated with the effector function of T lymphocytes (i.e., their ability to produce IFN-gamma). This does not end the list of potential factors that become useful in qualification of cancer patients for immunotherapy. There remains a need to search for new and perfect predictive factors for immunotherapy.

## Introduction

The immune system is a key player in the efficient monitoring and destruction of cancer cells (1). Let us follow briefly how it works. The immune response cycle begins with the recognition of tumor antigens by antigen-presenting cells and presents them to T lymphocytes in the lymph nodes. Activated cytotoxic T lymphocytes (CTLs) migrate to peripheral tissues, actively seeking the antigen (2, 3). At the site of recognition, the intracellular cytotoxic proteins are released from the CTLs, and together with the nonspecific mechanisms provided by macrophages or NK cells, the cancer cells are eliminated (1–4). Why does this system fail in some cases? It seems that the reasons lie both on the side of insufficient immune system activation and the growing tumor tissue (2, 4). First, the immune checkpoints expressed on immune system cells [CTLA-4 on T regulatory cells, PD-1 on T lymphocytes, PD-L1 on tumor-infiltrating immune cells (IC), e.g., macrophages] play a crucial role in maintaining a balance between immune system overactivation and extinguishing its action (5, 6). Whereas tumor cells (TC) that expressed the PD-L1 molecule could very effectively block the activity of PD-1-positive T lymphocytes. Keeping this in mind, how could we suppress the inhibiting activity of cancer cells and restore the cytotoxic activity of T lymphocytes? It is time for immunotherapy.

From the moment that Professor James Allison and Professor Tasuko Honjo discovered the checkpoint molecules, the next step was to create specific monoclonal antibodies that, by blocking these molecules, restore immune system activity (7, 8). Since this moment, immunotherapy with immunological checkpoint inhibitors (ICIs) has revolutionized cancer treatment, especially for patients without actionable driver mutations (8, 9). In 2016, the American Society of Clinical Oncology named immunotherapy as a top cancer advance of the year.

In the field of lung cancer immunotherapy, two groups of ICIs are widely used. Anti-PD-1 antibodies include pembrolizumab and nivolumab and block the PD-1 receptor on the lymphocyte surface. Anti-PD-L1 antibodies include atezolizumab, durvalumab, and avelumab and block the ligand for the PD-1 molecule on TC and on tumor-infiltrating IC (8–11). Now, one of the most important questions tormenting oncologists is how to choose patients with lung cancer who will benefit the most from immunotherapy? The solution to this problem is to choose the right and most sensitive biomarker (12, 13). At present, the only validated biomarker with a qualification for cancer patients for ICIs is the percentage of TC and/or IC with PD-L1 expression (12–14). Moreover, tumor mutational burden (TMB) and microsatellite instability assay also have a predictive value in qualification for immunotherapy (14–16). However, one should remember the availability of tissue material from cancer patients. What should we do if we cannot collect the cancer cells, or if the tumor is heterogeneous and we only have a small biopsy of tumor tissue? Does the determination of selected markers in the blood serum or plasma of cancer patients give reliable results and indicate those who should be treated with ICIs? Should we make every effort to reobtain tissue material?

In this article, we focus on the advantages and disadvantages of all biomarkers that are approved or were tested in clinical trials and that could be used in qualification of cancer patients for immunotherapy.

## Biomarkers Related to Tumor Tissue

### PD-L1 Expression

Immunohistochemical (IHC) testing for PD-L1 expression has become standard in the diagnosis of predictive factors in lung cancer (15–17). IHC is a relatively simple technique that is not related to any major problems. In many prospective trials, the efficacy of ICIs over standard chemotherapy as first-line therapy is demonstrated in patients with TC positive for PD-L1 (15–17). The KEYNOTE-024 study led to pembrolizumab registration in patients with metastatic non-small cell lung cancer (NSCLC) with expression of PD-L1 on ≥50% of TC (18). In this study, a significant increase in overall survival (OS) was observed in patients receiving pembrolizumab compared to patients treated with chemotherapy (18).

Moreover, based on the risk–benefit profile depicted in the KEYNOTE-042 study, pembrolizumab monotherapy can be extended by FDA registration (but not by the European Medicines Agency registration) as first-line therapy to patients with locally advanced or metastatic NSCLC without sensitizing *EGFR* or *ALK* alterations and with low PD-L1 expression (≥1%) as determined by an FDA-approved test (19).

However, advanced NSCLC patients, regardless of PD-L1 expression on TC, benefited from first-line combination therapy with platinum-based chemotherapy and pembrolizumab (KEYNOTE-189 and KEYNOTE-407 studies) (18–21). Regarding 2nd-line treatment, significantly longer survival was observed in locally advanced or advanced NSCLC patients receiving nivolumab (CheckMate-017 and CheckMate-057 studies) or atezolizumab (OAK study) compared with docetaxel regardless of PD-L1 expression although it should be noted that greater benefits were observed in patients with higher percentages of TC with PD-L1 expression (22–24). Pembrolizumab in the 2nd-line treatment could be used in patients with ≥1% of TC with PD-L1 expression (KEYNOTE-010 study) (25, 26). It should be mentioned that significant clinical efficacy of maintenance durvalumab therapy was observed in locally advanced NSCLC patients who received concurrent chemoradiotherapy (PACIFIC trial) (27, 28). In this study, patients were enrolled regardless of PD-L1 expression, but *post hoc* subgroup analysis of progression-free survival (PFS) and OS showed significant clinical benefits from durvalumab therapy when PD-L1 expression was present on ≥1% of TC (27). Recently, the FDA approved three new therapeutic strategies for first-line therapy in metastatic NSCLC patients: atezolizumab in patients with ≥50% of TC with PD-L1 expression (Impower-110 study); combination therapy with nivolumab and ipilimumab in patients with ≥1% of PD-L1 positive TC (CheckMate-227 study); and combination therapy with nivolumab, ipilimumab, and chemotherapy in patients regardless of PD-L1 expression on TC (CheckMate-9LA study) (29–33).

Additionally, an important issue regarding the benefit from immunotherapy in PD-L1 negative patients was raised in the CheckMate 227 trial (30, 34). In the subset of PD-L1 negative tumors, a significantly stronger survival benefit was observed in patients treated with nivolumab plus ipilimumab compared with chemotherapy. However, direct comparison of OS in PD-L1 negative tumors between combined immunotherapy and chemo-immunotherapy was not performed; a higher response rate for chemo-immunotherapy rather than for the nivolumab plus ipilimumab combination (38% *vs.* 27%) was observed. Based on that, we could speculate that immunotherapy combination and chemo-immunotherapy regimens demonstrated similar efficacy in PD-L1 negative patients (30, 34).

The abovementioned important clinical trials that resulted in immunotherapy registration clearly indicate that PD-L1 expression was an important factor for stratifying patients to receive ICIs and to obtain clinical efficacy (14–16, 35). However, it is surprising that different therapies used in different lines are registered in patients with different PD-L1 status on TC. It should be considered whether PD-L1 expression is an ideal biomarker or whether is it associated with ambiguity and controversy. Among the many unsolved questions concerning PD-L1 expression, the following aspect should be mentioned: 1) use of different cutoff levels for the percentage of PD-L1 positive TC for different ICIs; 2) differences in testing platforms; 3) the heterogeneous expression of this molecule through the tumor; its dependence on the histological type of TC; and the history of treatment (chemotherapy and radiotherapy could change PD-L1 expression on TC).

The scoring system for PD-L1 expression is varied for each immunotherapeutic. There were two categories in clinical trials with pembrolizumab: ≥50% of TC with PD-L1 expression is considered sufficient although <50% was insufficient for qualification for first-line therapy (18, 21). Pembrolizumab could be administered as second-line therapy if PD-L1 expression was observed on ≥1% of TC and in the first line with low PD-L1 expression (≥1%) but only in the United States (18, 19, 21, 35). Unlike this, nivolumab and atezolizumab could be ordered irrespective of PD-L1 expression on TC in second-line therapy (22–24, 31). However, in clinical studies with nivolumab in the second line, patients were stratified for PD-L1 expression into 4 groups: <1%, ≥1%, ≥5%, and ≥10% of PD-L1 positive TC. An even more complex scale was adopted in the OAK study. The efficacy of atezolizumab was assessed in 4 groups based on the PD-L1 expression on TC and tumor-infiltrating IC. Percentages of PD-L1-expressing TC were as follows: TC3 ≥50%, TC2 ≥5% and <50%, TC1 ≥1% and <5%, and TC0 <1% and percentage of tumor area infiltration by IC were as follows: IC3 ≥10%, IC2 ≥5% and <10%, IC1 ≥1% and <5%, and IC0 <1% (24). In the PACIFIC trial, ≥25% and ≥1% of TC with PD-L1 expression was used for assessment of durvalumab therapy efficacy; however, clinical benefits were observed irrespective of PD-L1 expression (27, 28). One should also remember that effectiveness of different treatment lines is also associated with various methods of qualification for treatment based on PD-L1 expression assessment.

As was mentioned, testing for PD-L1 expression by the IHC technique is considered to be a standard in predictive factor diagnosis. Unfortunately, each clinical trial with different immunotherapeutics used different anti-PD-L1 antibody clones and a different commercially available platform for testing. Trials with nivolumab used a 28-8 antibody clone, studies with pembrolizumab used a 22C3 clone, studies with atezolizumab used a 142 clone, and trials with durvalumab used an SP263 clone. The epitopes for anti-PD-L1 binding are in the extracellular domain for 28-8 and 22C3 antibody clones, and those for SP142 and SP263 are in the cytoplasmic domain (12, 21–24, 33, 35, 36).

In previous years, two large studies concerning the specificity and sensitivity of all the anti-PD-L1 antibody clones were conducted (Blueprint-1 and Blueprint-2) (35, 37, 38). In both studies, three companion assays—with 22C3 (used for pembrolizumab), 28-2 (used for nivolumab), and SP263 (used for durvalumab) antibody clones—achieved comparable specificity and sensitivity. Clone SP142, used for atezolizumab, was found to be less sensitive (37–39).

A quite complicated situation was resolved in 2015. The FDA approved the IHC 22C3 pharmDx assay as a companion diagnostic for the identification of NSCLC patients for pembrolizumab therapy (11, 12, 18). Moreover, in 2017, an IHC assay using the SP263 antibody clone, previously used for PD-L1 testing in clinical trials with durvalumab, received the CE mark for its use in PD-L1 testing during qualification for pembrolizumab immunotherapy. Therefore, PD-L1 expression is no longer required to be tested with the 22C3 antibody although this test is still approved for diagnosis (14–16).

The last problem associated with PD-L1 diagnosis is the heterogeneity of its expression within the tumor and its variability observed between primary and metastatic sites (14, 15, 40). Mansfield and colleagues examined paired primary lung tumor tissue and metastatic brain tissue and demonstrated that many of the brain metastases significantly lacked PD-L1 expression even when it was present in the primary lung cancer specimens (40).

During qualification of NSCLC patients for ICI treatment, all the limitations related to PD-L1 expression assessment described above as well as the spatial and temporal heterogeneity of the tumor microenvironment should be kept in mind.

### Tumor Mutational Burden

Cancer cells can be recognized by the immune system if there are tumor-specific antigens on their surface, and these arise as a result of somatic mutations in TC (14–16, 41, 42). During TC transformation, their genetic materials are very unstable, and gene reparation does not always occur properly. The total number of mutations within a tumor genome counted per coding area of a tumor genome is defined as the TMB (39). A higher number of somatic mutations causes an increased number of neoantigens, which translates into increased immunogenicity of such tissues (39). Cancer tissues with high TMB are thought to be more sensitive to immunological checkpoint inhibitors. Therefore, TMB could be a potential important biomarker in qualification to ICI therapy. Indeed, it has been used in numerous clinical studies (39, 41, 42).

The first studies that indicated its predictive value related to second-line therapy were clinical trials with atezolizumab (24, 31, 33). The OAK and POPLAR studies assessed TMB in patients’ blood and used a different cutoff level for TMB: 10, 16, and 20 mutations (mut) per megabase (Mb). Both studies reported a positive correlation between the number of mutations and OS as well as PFS of patients treated with atezolizumab. Moreover, both studies used the same platform for TMB assessment (Foundation One) (24, 31, 33, 35, 36).

The CheckMate-227 study is extremely important for the use of TMB as a predictive marker. In this study, combination therapy with ipilimumab and nivolumab was administered in first-line settings for chemotherapy-naive stage IV or recurrent NSCLC patients (32, 43, 44). The cutoff level for TMB was estimated at 10 mut/Mb. A significantly higher OS (18.3 months) was observed for high TMB patients (≥10 mut/Mb) when compared with patients (12.7 months) with low TMB (<10 mut/Mb). Furthermore, the significant efficacy of immunotherapy observed in patients with high TMB was irrespective of PD-L1 expression on tumor cells. The benefit of combination immunotherapy was observed even in patients with high TMB and <1% of TC with PD-L1 expression (32, 43, 44).

This biomarker could serve as a more sensitive predictor of immunotherapy benefit than PD-L1 expression on TC. However, it does not happen because, as a biomarker, TMB has some strong limitations (44–46). First, different clinical studies used various cutoff levels for defining TMB, ranging from 10 to 15 mut/Mb in tissue and from 6 to even 20 mut/Mb in plasma-derived, cell-free DNA (44, 47). In some studies, the cutoff scale was two points and in others 3 points and some of the studies defined TMB as high, medium, or low. In this regard, there is no standard definition of TMB that could be used to determine the level of mutations in further studies. However, the CheckMate 568 study used combination therapy (nivolumab and ipilimumab in untreated advanced NSCLC patients) and demonstrated that there was no evidence of increased immunotherapy efficacy in patients with very high TMB (≥15 mut/Mb) compared to patients with high TMB (≥10 mut/Mb) (43).

Second, different platforms were used for TMB estimation and various genetic techniques, including whole genome sequencing (WGS), whole exome sequencing (WES), or comprehensive genomic sequencing (CGS). Some panels require parallel sequencing of a paired normal specimen to exclude germline variants from analysis; others remove germline variants from tumor sequencing results (39, 45, 46). A harmonization study of TMB determination in NSCLC samples using three different commercially available sequencing methods was conducted by Garido-Martin and colleagues. They suggested that wider sequencing for more accurate TMB assessment is needed to reduced misclassification (48).

Ultimately, different materials were tested from NSCLC patients: tissue biopsies or peripheral blood. It should be noted that, in both peripheral blood and tumor tissue, the percentage of results that could not be analyzed was relatively high (32, 47). In the CheckMate 227 study, which used tissue materials and the Foundation One CDx assay, the rejection rate was estimated at 47% of analyzed specimens (32). This suggests that, for reliable TMB testing, particularly good quality material is required. These facts translated into FDA registration of nivolumab in combination with ipilimumab in advanced NSCLC patients based on the results of the CheckMate 227 study. This combination therapy can be administered as first-line therapy in patients with ≥1% of TC expressing PD-L1. TMB in these patients does not need to be evaluated (11, 32, 39, 43).

Taken together, TMB has a notably great potential as a predictive biomarker. Indeed, further standardization of methods used in TMB assessment and systematic evaluation of TMB across different sequencing platforms should be undertaken before it is fully incorporated into clinics.

### Immunoprofile and Gene Expression Signature of Tumor Tissue

Studies carried out in many different cancers have proven that the presence of IC, especially tumor infiltrating T lymphocytes (TILs) in the tumor tissue, is associated with higher benefits from immunotherapy (14–16, 49, 50). These observations are widely described in patients with breast cancer or melanoma (49, 50). Moreover, using data regarding ICI effectiveness in various malignancies, it has been indicated that tumors have three immunoprofiles based on their immune system activation: 1) “hot” tumors, which are strongly infiltrated by T lymphocytes and with many inflammatory signals; 2) “cold” tumors, which have scant IC infiltration or inflammatory signs; and 3) tumors with immune exclusion, in which immune cells are at the periphery or within the stromal tissue (49–51).

Are similar divisions of cancerous tissue described in NSCLC, and even more interestingly, has analysis of the immune system in cancerous tissue ever been used as a predictor for ICI efficacy?

In the case of lung cancer, it seems that a clear division into three types of tumor tissue has never been used prospectively as an ICI predictor in clinical trials. Rather, the immune gene signature profile, particularly those associated with IC activation (e.g., INF-γ signaling), instead of immunological examination, is correlated with immunotherapy outcome (14, 15, 31). In the POPLAR trial, in which the efficacy of atezolizumab was compared with docetaxel in a second-line setting, retrospective analysis showed that significant improvement in patient survival was associated with a high expression of the interferon-γ gene and genes associated with the T-effector activation (defined by *CD8A*, *GZMA*, *GZMB*, *IFN-γ*, *EOMES*, *CXCL9*, *CXCL10*, and *TBX21* gene expression) (31). All these genes had high co-expression in tested tumor specimens, which have been previously associated with activated T cells, immune cytolytic activity, and interferon-γ expression (31). It is obvious that tissue with high expression of these mentioned genes meets the criteria for a “hot, inflamed tumor,” but it was based on genetic, not immunological, examination. The “hot” tumors are associated with denser PD-1-positive T lymphocytes infiltration with a preexisting primed immune response, and they are more likely to respond to an anti-PD-1 or anti-PD-L1 blockade used as monotherapy (31, 49).

Are these relationships also observed when we use a combination of immunotherapy, which seems to have a great importance for the future? In the IMpower 150 study, among patients with no PD-L1 expression on TC, with low expression of the genes responsible for T-effector activation, and with liver metastases, a significantly longer median PFS was observed in the group of patients receiving combination therapy (atezolizumab, bevacizumab, chemotherapy) than in patients receiving only bevacizumab with platinum doublets (33). However, when tissue samples were qualified as “inflamed,” more benefits from combination therapy were observed in patients carrying “hot” tumors.

The greater efficacy of immunotherapy in patients with “inflamed” than in patients with “non-inflamed” tumors seems to be well documented in the literature (50, 51). What about the tumor, where the preexisting immune response is located at the invasive tumor margin? Lung cancer trials are rather scarce on this observation. However, it seems, based on melanoma trials, that tumor regression after therapeutic PD-1 blockade requires tumor infiltration by CD8-positive cells (50). Pretreatment samples obtained from melanoma patients who later responded to pembrolizumab therapy showed a higher number of CD8-positive, PD-1-positive, and PD-L1-expressing cells at the invasive tumor margin and inside tumors (52).

Currently, none of anti-PD-1 or anti-PD-L1 antibodies could be administered based on the immunological status of the tumor tissue (11, 12). However, immunological analysis or estimation of the gene expression profile in cancer tissue could be considered to be a reliable biomarker in the prospective qualification for immunotherapy. What should be chosen for the future direction? Immunological analysis of the existing immune response in cancer tissue could be added into the basic pathomorphological diagnosis. This is a relatively quick and inexpensive technique, but it requires several serially cut tissue specimens. Molecular analysis of cancerous tissue evaluated by gene expression could be carried out simultaneously in one tissue fragment, but it requires a specific molecular platform as a microarray. However, for both these methods, a bright future is ahead, and expanding the benefits from immunotherapy based on profiling of immune and genetic characteristic of tumors is possible, but prospective validation is still needed.

### Genetics Biomarkers

Very recent studies indicate that some genetic abnormalities could be considered as predictive biomarkers for immunotherapy. *STK11/LKB1*-inactivating mutations have been significantly linked to a primary resistance to PD-1 inhibitors (53). Patients harboring the *STK11* mutation had a significantly lower expression of PD-L1 molecules on TC and higher TMB score and no clinical benefits were observed when they received immune checkpoint inhibitors with a median survival of only 6 months. Moreover, the presence of this mutation strongly correlated with the low immune cell infiltration within the tumor tissue (53). That indicates that an *STK11*-positive tumor could be defined as the cold type, which directly translates into poor immunogenicity. Quite often the *STK11* mutation significantly coexists with the *KRAS* and *KEAP1* mutations in cancer patients. The kelch-like ECH-associated protein 1 (Keap1)-nuclear factor erythroid 2-related factor 2 (Nrf2) intracellular pathway is defined as a factor regulating genes related to the cellular protective response as well as to resisting the action of chemotherapy drugs (54, 55). Malfunctioning of *Nrf2* and *Keap1* genes has been observed in lung cancer, and it is possible that they are associated with tumor progression, cytoprotection, and poor prognosis. However, clinical implementation of Nrf2 inhibitors in patients with advanced NSCLC may be a useful therapeutic approach for patients harboring *KEAP1-NRF2* mutations, increasing the chance for clinical response (54, 55).

In the context of genetic markers, their determinations seem to be of great importance in predicting resistance to immunotherapy. T cell–mediated cytotoxicity could be deregulated by mutations in genes involved in chromatin remodeling pathways. The mutations in the *SWI/SNF* (SWItch/sucrose nonfermentable) complex as well as in the *PBAF* complex (PBRM1, ARID2, and BRD7) regulate the chromatin opening for the IFN pathway in TC, resulting in an increased resistance to lymphocyte cytotoxicity. This resistance can be reversed by *PBRM1* as well as *ARID1A* gene inactivation (56).

## Biomarkers Related to Peripheral Blood

One could imagine an ideal situation in which biomarker determinations for ICI qualification is performed in a material as easily accessible as peripheral blood. This is already established for patients progressing on EGFR tyrosine kinase inhibitors when the presence of the Thr790Met mutation in the *EGFR* gene is examined in peripheral blood for osimertinib qualification (57, 58). Is there any chance that biomarkers tested in peripheral blood would indicate a group of patients benefiting more from immunotherapy? To date, most published analysis of peripheral blood biomarkers has been tested retrospectively (12–14). However, it seems that they have a lot of information about the activity of the immune system in cancer patients.

## Peripheral Blood Soluble Biomarkers

The most investigated serum soluble biomarker is blood tumor mutational burden (bTMB), estimated by commercial platforms (e.g., the FoundationOne CDx assay) in cell-free DNA (not in peripheral blood circulating cancer cells) (59, 60). The most recognizable studies that determined bTMB were POPLAR, OAK, and CheckMate 227 (24, 31, 32). Gandara et al. described a novel, technically robust, blood-based assay to measure bTMB based on hybridization-capture methodology, which is distinct from tissue-based approaches (47). First, they showed positive correlation between blood and tissue TMB in advanced NSCLC patients treated with second- or third-line immunotherapy included in the POPLAR trial. The cutoff points for bTMB that significantly correlated with outcomes of patients treated with atezolizumab were confirmed in the OAK study (24, 31, 47). They found that significantly longer PFS in atezolizumab-treated patients was associated with higher bTMB, and the definition of high TMB was estimated as ≥16 mut/Mb (24, 31, 47). Both studies have undoubtedly shown that bTMB could be a predictive biomarker for ICI qualification. Notwithstanding patients with bTMB ≥16 mut/Mb showed benefits from combination immunotherapy (tremelimumab plus durvalumab) in the MYSTIC clinical trial. In addition, patients with TMB ≥10 mut/Mb in tissue usually had TMB ≥16 mut/Mb in their blood serum (61, 62). The MYSTIC trial was a negative study in which the clinical benefit of combination therapy over chemotherapy was not demonstrated, but this study allowed a prospective determination of the TMB ≥10 mut/Mb cutoff threshold for the CheckMate 227 study.

## Peripheral Blood–Soluble PD-1 and PD-L1

The soluble form of PD-L1 (sPD-L1) is usually undetectable in the plasma of healthy people (63). However, detection of sPD-L1 is associated with a poor prognosis in various cancers. Moreover, a high level of sPD-L1 is associated with systemic inflammation and with activation of a nonspecific immune response (63). Taken together, this factor could be considered a predictive marker for immunotherapy qualification. So far, only one study has looked at the use of sPD-L1 as a prognostic factor in NSCLC patients. Interestingly, in the group of EGFR-mutated NSCLC patients, the increased level of sPD-L1 during erlotinib therapy was associated with a better prognosis. It is remarkably interesting because it is obviously known that patients with driving mutations do not receive benefits from immunotherapy (63). Meyo MT et al. showed the potential predictive role of soluble sPD-1 and sPD-L1 expression examination in metastatic NSCLC patients receiving nivolumab therapy (63). After two cycles of nivolumab therapy, increased sPD-1 levels, compared with the baseline value, independently correlated with longer PFS (adjusted HR=3.32; p=0.013) and OS (adjusted HR=0.33; p=0.006), and this relation was not seen when analyzing other soluble biomarkers (e.g., sCD40L, sCD44, or VEGFA). The authors proposed that composite biomarker analysis using sPD-1 and sPD-L1 could predict nivolumab efficacy (63). Zhang J et al. determined the expression of circulating PD-L1 in samples taken from 109 advanced NSCLC and 65 healthy patients (64). The results were analyzed with the association between clinicopathologic features and prognosis. First, Zhang J et al. showed higher PD-L1 expression in advanced NSCLC patients compared with the healthy controls (p<0.001). Moreover, high PD-L1 expression was positively correlated with shorter OS compared with low expression (18.7 vs. 26.8 months, p < 0.001). The presented results may give hope for the future use of sPD-1 or sPD-L1 determinations as prognostic factors (64). Moreover, the high levels of these molecules were related to intense inflammation and unspecific immune response activation, which is also considered to be a positive factor from immunotherapy benefits.

## Plasma-Circulating Tumor DNA and PD-L1-Carried Exosomes

When we discuss the determination of predictive factors in a patient’s blood serum, do not forget about the possibility of examining plasma-circulating tumor DNA (ctDNA). Ricciuti B et al. showed that assessment of plasma ctDNA would enable early detection of response to immunotherapy in NSCLC patients even before radiological examinations (65). Advanced NSCLC patients treated with first-line pembrolizumab +/- platinum doublet chemotherapy were enrolled in this study, and plasma samples were collected prior to starting therapy and serially during treatment. ctDNA was analyzed by NGS using targeted amplification of hot spots (65). Ricciuti B et al. showed that median PFS (mPFS) and median OS (mOS) were significantly longer among patients with low ctDNA levels tested at baseline compared with those with an increase in ctDNA (mPFS: 13.7 vs. 3.4 months, HR:0.20, P < 0.01; mOS: 32.8 vs. 14.7 months, HR:0.06, P < 0.01) (65). Similarly, Jia N et al., in a study of metastatic colorectal cancer patients treated with a first-line chemotherapy regimen, showed that changes in ctDNA determined by various techniques may have a strong predictive value for the assessment of patients’ responses (66). Taken together, these results suggest that rapid changes in ctDNA could be applied as an early pharmacodynamic biomarker of response or resistance to immunotherapies (65, 66). Unfortunately, today it seems that the potential related to ctDNA examination is not fully utilized in the clinic.

The presence of exosomes that carry PD-L1 molecules on their surface may be another predictive factor of response to anti-PD-1 or anti-PD-L1 antibody therapy (67, 68). Exosomes, which are extracellular vehicles, are produced by cancer cells and released into the tumor microenvironment. In malignant melanoma patients, the high level of PD-L1-carried exosomes positively correlated with IFN-γ and indicated a high-level stimulation of an adaptive response in the early course of the disease (67, 68). Studies reported by Chen G et al. indicated that the level of PD-L1-carried exosomes could be a predictive factor that stratifies patients qualified for immunotherapy into two groups: responders with a high level of PD-L1-carried exosomes and nonresponders with a low level of these molecules (69). However, there are two important issues that should be kept in mind. First, there are methodological difficulties in examining the level of exosomes. At present, this test is not performed as a routine predictive factor but as research used for scientific purposes. Second, we should consider whether patients with high levels of PD-L1-carried exosomes should be treated with anti-PD-L1 or anti-PD-1 monoclonal antibodies or with antibodies specifically blocking PD-L1 exosomes or with combination therapy that can lock both of these points. These questions need to be answered in the future.

## Peripheral Blood Cellular Biomarkers

We should consider the use of conventional signs of inflammation tested in peripheral blood, such as LDH, C-reactive protein, or IL-6 concentration. Current data indicate only retrospective analysis of inflammatory-associated factors in NSCLC patients who received immunotherapy (12–15).

The simple analysis of selected peripheral blood parameters provides basic but particularly important information about the patients’ immune system status. However, it is problematic to talk about predictive factors based on simple blood testing. This testing could be a great source of information about rapid progression during ICI therapy (70). For instance, the neutrophil-to-lymphocyte ratio (NLR), which could be simply calculated from a complete blood testing report, attracted a lot of interest regarding detection of rapid progression during ICI treatment (71, 72). The studies conducted in the tumor microenvironment of different solid cancers demonstrated that increased neutrophil infiltration should be considered as a factor promoting tumor progression (70–72). The NLR has also been studied in NSCLC patients. Takeda and colleagues reported that NLR could distinguish between nonresponders and responders to nivolumab therapy at an early stage of treatment, which is crucial for rapid progression diagnosis (73). The results show that low NLR (<5) after 4 weeks of nivolumab administration was significantly associated with higher median PFS compared to patients with NLR≥5. Takeda et al. indicate that the expression of these markers fluctuates dramatically during treatment; therefore, repeated evaluation is essential (73). Liu J and colleagues explored the systemic immune-inflammation index (SII), which combines NLR and platelet-to-lymphocyte ratio (PLR) (74). SII is a novel inflammatory marker, and it is considered to be an independent risk factor for the development of solid cancer. Low SII, NLR, and PLR were significantly associated with higher median PFS for metastatic NSCLC patients treated with nivolumab as second- or third-line treatment (74).

A literature review shows that more effort is made to retrospectively assess baseline peripheral blood biomarkers and associate them with clinical outcomes in NSCLC patients treated with immunotherapy (71, 72). Tanizaki and colleagues evaluated the relationship between survival and peripheral blood parameters measured before nivolumab initiation, including absolute neutrophil count (ANC), absolute lymphocyte count (ALC), absolute monocyte count, absolute eosinophil count (AEC), serum C-reactive protein, and lactate dehydrogenase concentrations (70). Low ANC, high ALC, and high AEC were significantly and independently associated with both higher mPFS and mOS in multivariable analysis. Additionally, the patients with only one positive predictive factor had a significantly worse outcome than those with two or three factors. All patients with ≥50% PD-L1 expression on TC had at least two favorable factors (70). This may suggest that high PD-L1 expression on TC influences systemic inflammation parameters, and combined analysis of those parameters could better predict response to ICI therapy. Unfortunately, the studies have been conducted on a small group of patients, and future validation is still necessary.

Some studies report that clinical benefits could be predicted based on regulatory T cell examination in patients with melanoma (75). Moreover, a high percentage of myeloid-derived stem cells (MDSCs) in peripheral blood was negatively correlated with the clinical benefit for ipilimumab-treated patients (75). Regrettably, those examinations are of very marginal importance.

## Conclusion

Unquestionably, the effectiveness of immunotherapy has been proven in many clinical studies and documented by numerous registration approaches. Nevertheless, the issue that still raises some concerns is the use of appropriate biomarkers for qualification of cancer patients to immunotherapy. The presented work summarizes the most important information about biomarkers that could be used in the clinic. All this information is summarized in **Table 1**, but be aware of the following points. First, NSCLC patients should always be qualified for immunotherapy in regard to the registration summary of each immunotherapeutic based on the predictive factors that are dedicated to them. Currently, expression of the PD-L1 molecule on TC for immunotherapy of advanced NSCLC patients is the only predictive factor validated in prospective clinical trials. However, recently, new immunotherapeutic registrations are based on TMB as a predictive factor although this has not been validated as deeply as PD-L1 expression.

**Table 1:** Summary of the most important advantages or disadvantages of the described biomarkers used in qualification of NSCLC patients to immunotherapy.

Biomarkers related to tumor tissue	
**PD-L1 expression on tumor cells or tumor-infiltrating-immune cells**	only validated biomarker in many prospective trials positivity for PD-L1 expression was defined using different values of PD-L positive tumor cells percentage evaluation of percentage of tumor area infiltrated with immune cells expressing PD-L1 is extremely difficult and useless expression was tested with different platforms tumor tissue could demonstrate heterogenicity for PD-L1 expression PD-L1 expression could depend on the histological type of tumor cells and patients’ history of treatment
**Tumor mutational burden**	proven to be a valuable factor in combination therapy regardless of PD-L1 expression various cutoff levels for defining TMB level different platforms were used for TMB estimation different samples were tested for TMB with high rejection rate related to tumor samples
**Immunoprofile of tumor tissue**	has never been used in prospective trials with ICI therapy immunological analysis could be added into the basic pathomorphological diagnosis relatively quick and inexpensive technique requires several serially cut tissue specimens
**Gene expression signature**	interferon-γ gene signature retrospectively demonstrated predictive value for ICIs therapy molecular analysis could be carried out simultaneously in one tissue specimen required specific molecular platform
**Mutations in immunotherapy-resistance genes: *STK11*, *KEAP1***	estimated by NGS technique or single-gene testing significantly associated with poorer OS lacks prospective validation in clinical trials
**Biomarkers related to peripheral blood**	
**Peripheral blood-soluble biomarkers**	
**Blood TMB**	positive correlation between blood and tissue TMB was shown determined in the most easily accessible blood samples has never been used prospectively as an ICI therapy predictor
**Soluble PD-1, PD-L1**	increase level at baseline correlated with ICI benefit used as additional research used only as retrospective factors
**ctDNA and PD-L1-carried exosomes**	rapid changes in ctDNA as an early pharmacodynamic biomarker of response or resistance to ICIs used as additional research used only as retrospective factors
**Inflammation parameters tested in blood analysis: LDH, C-reactive protein, IL-6 plasma, or serum concentration**	no impact on ICI effectiveness used as additional research usually in scientific research
**Peripheral blood cellular biomarkers**	
**Neutrophil-to-lymphocyte ratio (NLR)**	simple analysis performed during completed blood testing could be a great source of information about the rapid progression during ICI therapy it fluctuates dramatically during treatment; repeated evaluation is essential
**Systemic inflammation parameters: absolute neutrophil count (ANC), absolute lymphocyte count (ALC), absolute monocyte count, absolute eosinophil count (AEC)**	has never been used prospectively as a ICI predictor significantly and independently associated with PFS and OS systemic inflammation parameters combined with PD-L1 expression could better predict response for ICIs therapy

Based on the clinical trials conducted so far, we could conclude that one perfect predictive biomarker does not exist, and during qualifying cancer patients for immunotherapy, at least two biomarkers should be taken into account. One should remember that the immune system is a complex network of intercellular interactions, and it is difficult to talk about a single factor that determines its activity. Unfortunately, it seems that we will never achieve the situation that occurs for molecularly targeted therapy, in which one driving mutation affects treatment effectiveness. In addition, the situation with biomarkers could be more complicated when new immunotherapies targeting the remaining negative immune control points, anti-TIGIT or anti-TIM-3, are introduced in the clinic.

Second, we do not dismiss the possibility of using knowledge about additional biomarkers. We should also remember that many biomarkers that have not been registered so far can greatly facilitate monitoring of immunotherapy effectiveness. They are imperfect indeed but still could be important to prevent rapid tumor progression or for identification of the site effects of immunotherapy.

## Author Contributions

KW-K—literature review, preparing the manuscript, editing the manuscript TK—literature review, preparing the manuscript. All authors contributed to the article and approved the submitted version.

## Conflict of Interest

The authors declare that the research was conducted in the absence of any commercial or financial relationships that could be construed as a potential conflict of interest.
